# Analysis of Methylation Dynamics Reveals a Tissue-Specific, Age-Dependent Decline in 5-Methylcytosine Within the Genome of the Vertebrate Aging Model *Nothobranchius furzeri*

**DOI:** 10.3389/fmolb.2021.627143

**Published:** 2021-06-16

**Authors:** Gordin Zupkovitz, Julijan Kabiljo, Michael Kothmayer, Katharina Schlick, Christian Schöfer, Sabine Lagger, Oliver Pusch

**Affiliations:** ^1^ Center for Anatomy and Cell Biology, Medical University of Vienna, Vienna, Austria; ^2^ Department of Life Science Engineering, University of Applied Sciences Technikum Wien, Vienna, Austria; ^3^ City of Vienna Competence Team Aging Tissue, Vienna, Austria; ^4^ Department of General Surgery, Comprehensive Cancer Center Vienna, Medical University of Vienna, Vienna, Austria; ^5^ Unit of Laboratory Animal Pathology, University of Veterinary Medicine Vienna, Vienna, Austria

**Keywords:** aging, epigenetics, DNA methylation, DNA hydroxymethylation, epigenetic aging clock, African turquoise killifish, *Nothobranchius furzeri*

## Abstract

Erosion of the epigenetic DNA methylation landscape is a widely recognized hallmark of aging. Emerging advances in high throughput sequencing techniques, in particular DNA methylation data analysis, have resulted in the establishment of precise human and murine age prediction tools. In vertebrates, methylation of cytosine at the C5 position of CpG dinucleotides is executed by DNA methyltransferases (DNMTs) whereas the process of enzymatic demethylation is highly dependent on the activity of the ten-eleven translocation methylcytosine dioxygenase (TET) family of enzymes. Here, we report the identification of the key players constituting the DNA methylation machinery in the short-lived teleost aging model *Nothobranchius furzeri.* We present a comprehensive spatio-temporal expression profile of the methylation-associated enzymes from embryogenesis into late adulthood, thereby covering the complete killifish life cycle. Data mining of the *N. furzeri* genome produced five *dnmt* gene family orthologues corresponding to the mammalian *DNMTs* (*DNMT1, 2, 3A,* and *3B*). Comparable to other teleost species, *N. furzeri* harbors multiple genomic copies of the *de novo* DNA methylation subfamily. A related search for the DNMT1 recruitment factor *UHRF1* and *TET* family members resulted in the identification of *N. furzeri uhrf1, tet1, tet2,* and *tet3*. Phylogenetic analysis revealed high cross-species similarity on the amino acid level of all individual dnmts, tets, and uhrf1, emphasizing a high degree of functional conservation. During early killifish development all analyzed *dnmts* and *tets* showed a similar expression profile characterized by a strong increase in transcript levels after fertilization, peaking either at embryonic day 6 or at the black eye stage of embryonic development. In adult *N. furzeri,* DNA methylation regulating enzymes showed a ubiquitous tissue distribution. Specifically, we observed an age-dependent downregulation of *dnmts*, and to some extent *uhrf1,* which correlated with a significant decrease in global DNA methylation levels in the aging killifish liver and muscle. The age-dependent DNA methylation profile and spatio-temporal expression characteristics of its enzymatic machinery reported here may serve as an essential platform for the identification of an epigenetic aging clock in the new vertebrate model system *N. furzeri.*

## Introduction

Both, genetic and non-genetic factors impact the aging process. Multiple studies in model organisms and humans have suggested that epigenetic alterations play a drastic role in the age-associated physiological decline and disease. It has become increasingly clear that rather than being genetically predefined, lifespan is largely epigenetically determined (López-Otín et al.,). By definition, epigenetics represents the reversible and heritable mechanisms–passed on through either mitosis or meiosis–occurring without changing the underlying DNA sequence (Berger et al., 2009). Epigenetic changes causing deteriorated cellular functions during aging occur at various levels, including histone modifications, altered expression of non-coding RNAs and alterations in DNA methylation, the latter being the most studied mechanism (Booth and Brunet 2016). In eukaryotes, the addition of a methyl group generating 5-methylcytosine (5mC) mainly occurs in the context of CpG dinucleotides. DNA methylation patterns are established by the action of the opposing enzymatic activities of DNA methyltransferases (DNMTs) and the ten-eleven translocation (TET) family of dioxygenases that apply and remove DNA methylation, respectively (Bird 1986; Tahiliani et al., 2009). DNA methylation is one of the earliest described epigenetic marks and a large body of experimental evidence links it to many forms of stable epigenetic repression, such as genomic imprinting, X chromosome inactivation in females and silencing of repetitive DNA (Schübeler 2015). Notably, several traditional aging models such as *Drosophila melanogaster*, *Caenorhabditis elegans*, and the fungus *Saccharomyces cerevisiae* exhibit virtually no 5mC and are therefore not well suited for studying the role of 5mC methylation in organismal lifespan (Booth and Brunet 2016). Despite the fact that DNA methylation is in general a stable epigenetic mark, a phenomenon described as “epigenetic drift” reduces the stringency of DNA methylation maintenance over lifetime (Egger et al., 2004). This non-directional DNA methylation drift involves both hyper- and hypomethylation events on genomic DNA. As a consequence, sparsely methylated areas such as promoter-associated CpG islands locally gain DNA methylation with age whereas highly methylated regions lose methylation with age. The functional consequence of this epigenetic erosion results in a global loss of DNA methylation in advanced life and a blurring of the epigenetic DNA methylation landscape (Illingworth and Bird 2009; Hannum et al., 2013; Weidner et al., 2014). Strikingly, the methylation level of individual CpG dinucleotides in the genome are highly associated with age and collections of specific methylation sites can serve as an accurate prediction system of chronological age. This gradual accumulation of differential DNA methylation, unlike DNA methylation entropy referred to as epigenetic drift, are common between individuals and sometimes even tissues and comprise an “epigenetic clock.” So far, DNA methylation based epigenetic clocks have been successfully developed only in mammals including humans, mice, whales, dogs, and wolves (Horvath and Raj 2018).

In the present study, we analyzed the DNA methylation machinery during the entire life cycle of the new vertebrate aging model *Nothobranchius furzeri* (Genade et al., 2005) with regard to its evolutionary conservation, transcriptional expression profiles and changes in global DNA methylation and hydroxymethylation patterns. Initially collected in 1968 in Zimbabwe, *N. furzeri* represents a naturally short-lived vertebrate populating temporary ponds in South-East Africa. Notably, with a lifespan of only 4–6 months under laboratory conditions (6–10 times shorter than the lifespan of mice and zebrafish, respectively), the turquoise killifish currently constitutes the shortest-lived vertebrate that can be bred in captivity (Genade et al., 2005; Terzibasi et al., 2007). Despite its short lifespan, the fish shows typical aging-related phenotypes such as physiological and cognitive decay, expression of aging-related biomarkers, a decline in fertility, sarcopenia, and cancerous lesions (Di Cicco et al., 2011; Kim et al., 2016). Importantly, *N. furzeri* responds to age-effecting environmental stimuli such as caloric restriction (Terzibasi et al., 2009), a resveratrol-rich diet (Valenzano et al., 2006b), and temperature (Valenzano et al., 2006a). The turquoise killifish reached another critical milestone as a novel model system when its genome was sequenced in 2015 (Reichwald et al., 2015; Valenzano et al., 2015). Recently, techniques for microinjection and production of transgenic fish lines (Valenzano et al., 2011; Hartmann and Englert 2012; Allard et al., 2013) and a toolbox for precise genome-editing (Harel et al., 2015) were successfully implemented. These advances elevate *N. furzeri* to an ideally suited research organism to systematically model and study vertebrate aging and aging-related diseases.

## Materials and Methods

### Killifish Care and Maintenance

All animal experiments were performed on the inbred *N. furzeri* strain GRZ (generously provided by Dario Valenzano, Max Planck Institute for Biology of Aging, Cologne, Germany). The fish colonies were raised in the killifish facility at the Medical University of Vienna (MUV). General fish maintenance and husbandry was performed according to standardized protocols (Genade et al., 2005; Zupkovitz et al., 2018a). Adult male animals subject to further analyses (qRT-PCR analysis and global DNA methylation/hydroxymethylation quantification) at time points week 5, 11, 15, 19 were kept in a stand-alone overflow system housing a single fish per 2.8 L tank. Killifish were euthanized with MS-222 and incubated on ice for 5 min before dissection and organ harvesting. To prevent undesirable biases due to circadian rhythms and/or feeding regiments, individual killifish were sacrificed in a fasted state at comparable hours in the morning. For downstream molecular biology analyses, harvested organs were snap-frozen in liquid nitrogen and stored at −80°C.

### Survival Curve

To determine survival rates of the *N. furzeri* strain GRZ housed in the MUV fish facility, age-dependent mortality data of ten consecutive hatches consisting of 335 female and 283 male fish (total 618) were combined. Survival data of fish that were used for strain maintenance were not considered. Experimental set up was designed as previously described (Kabiljo et al., 2019). In brief, at the age of 5 weeks where the sex of individual killifish was conclusively identified, mixed-sex groups were set up housing eight animals per 25 L tank. Five weeks of age was additionally used as an experimental starting point for all analyses described in this report. Tanks were surveyed for deceased killifish and animal counts were recorded two times per day. To obtain survival curves, animal numbers were calculated and depicted on a weekly scale without correcting for population density due to diminishing group size. Survival is shown as the percentage of living killifish at a particular age per total number of fish.

### Sampling of Embryonic Stages and Newly Hatched Fish

For gene expression profiling of the killifish DNA methylation machinery during embryonic development and early life cycle, we included six different time points: 1–2 cell stage, embryonic day 3 (E3), embryonic day 6 (E6), black and golden eye stage and fish 24 h post hatching (D1). Sampling of developmental stages was monitored by microscopic observation of morphological criteria. For total RNA isolation the following numbers of embryos per biological replicate were sampled (1–2 cell: 200 eggs; E3 and E6: 100 eggs; black and golden eye: 50 eggs; hatched D1: 30 fish).

### Sequence Comparison and Phylogenetic Analysis

The protein sequences for *N. furzeri* methylation machinery members were downloaded from the NFINgb genome browser (http://nfingb.leibniz-fli.de) (Reichwald et al., 2015) whereas the sequences for all other analyzed species were obtained from ENSEMBL (http://www.ensembl.org). We considered the following species: human (*Homo sapiens*)*,* mouse (*Mus musculus*), zebrafish (*Danio rerio*)*,* medaka (*Oryzias latipes*)*,* and coelacanth (*Latimeria chalumnae*). After having generated a high quality multiple alignment with the Muscle program (Edgar 2004), the phylogenetic tree was computed using the Maximum-Likelihood algorithm applying the LG substitution matrix with the PhyML program (Guindon et al., 2009). In the last step, amino acid sequence similarity assessed by pairwise sequence comparison of full-length proteins was derived from the alignment using Geneious 11.0.3 software (Supplementary Table 2). The accession numbers for all used protein sequences are listed in Supplementary Table 1.

### RNA Isolation and Quantitative Real-Time RT-PCR Analysis

RNA isolation from *N. furzeri* embryos and tissues using TRI reagent (Sigma-Aldrich) and quantitative Real Time RT-PCR analysis were performed as described (Zupkovitz et al., 2018b). Total RNA was isolated from either snap-frozen whole embryos at different stages or brain, liver and muscle samples of adult fish following the manufacturer’s instructions. One microgram of isolated RNA was reverse transcribed with the iScript cDNA synthesis kit (Bio-Rad) and Real-time RT-PCRs were performed using the CFX384 Well qPCR Detection System (Bio-Rad) and Blue S’Green qPCR Kit (Biozym). TATA binding protein (*tbp*) housekeeping gene expression was used for normalization. All used primer sequences are listed in Supplementary Table 3. All expression raw values obtained from the Real-Time RT-PCR analysis (normalized to *tbp*) are listed in Supplementary Table 4. Real-time PCR experiments were initially analyzed with the CFX Maestro software. Graphical output and statistics were performed with Prism software (GraphPad). Statistical significances were calculated with one-way ANOVA implementing Tukey’s multiple comparisons. *p*-values were calculated employing Prism software and standard deviation (SD) is shown. *p* > 0.05; *: *p* ≤ 0.05; **p ≤ 0.01; ***: *p* ≤ 0.001; ****: *p* ≤ 0.0001.

### Global DNA Methylation and Hydroxymethylation Quantification

Genomic DNA from *N. furzeri* tissues was extracted using the Gentra Puregene Tissue Kit (Qiagen) according to manufacturer’s instructions and dot blots were done as previously described (Lagger et al., 2017). In brief, dot blots of genomic DNA and modified control oligonucleotides (unmethylated: oligo control, 5-methylated CpG: oligo 5mC, and 5-hydroxymethylated CpG: oligo 5hmC) were produced with Bio-Rad’s Bio-Dot Microfiltration apparatus according to manufacturer’s instructions. The control oligos (ordered from www.biomers.net) consisted of a synthetic sequence (49% GC content, 109bp in length) and T3 and M13-20 primer binding sites (underlined) with the following sequence: 5′-ATG​CTA​ATT​AAC​CCT​CAC​TAA​AGG​GAA​CTC​GAG​ACA​TCG​GAG​AAT​TCA​CAT​CAC​CGG​TGA​ATC​AGT​GCT​ACC​CGC​AAG​TGC​ACT​GGA​TCC​ACT​GGC​CGT​CGT​TTT​ACA​A-3′. Differentially methylated CpGs are highlighted in bold. Genomic DNA and oligonucleotide controls were denatured by boiling for 10 min at 100°C in 0.4 mM NaOH/10 mM EDTA in a total volume of 25 µl. Prior to spotting, DNA samples were neutralized by adding an equal volume of ice-cold 2 M ammonium acetate. Genomic DNA in technical duplicates (250 ng per well) and control oligonucleotides in a 1:2 dilution series (5 µM starting concentration) were spotted on a nitrocellulose membrane pre-soaked in 6x SSC buffer. After washing in 2x SSC, the membrane was crosslinked with a UV Stratalinker (Stratagene) and blocked in 5% non-fat dried milk powder in 0.1% Tween-20/1x TBS for 30 min at room temperature. The membranes were incubated with primary antibodies overnight at 4°C while rolling (5mC: 1:500 Eurogentec # BI-MECY-0100; 5hmC: 1:5.000 Active Motif # 39792). After incubation with secondary antibodies (anti-rabbit/anti-mouse IgG HRP conjugate, 1:10.000), 5mC and 5hmC signals were recorded using the ChemiDoc XRS + Imaging System (Bio-Rad) and analyzed with ImageLab Software (Bio-Rad). For normalization, the membranes were incubated with 0.02% methylene blue in 0.3 M sodium acetate pH5.2 for 5 min at room temperature, briefly destained in H_2_O and imaged as described above. Levels of 5mC and 5hmC were normalized to the respective methylene blue signal intensities and calculated as mean of three biological replicates per time point. Raw values of the dot blot quantification are listed in Supplementary Table 4. Statistical significances were calculated with one-way ANOVA and multiple comparisons were performed by comparing the means of W11, W15, and W19 to W5 set to 1. ns: *p* > 0.05; *: *p* ≤ 0.05; **p ≤ 0.01; ***: *p* ≤ 0.001; ****: *p* ≤ 0.0001.

## Results

### Phylogenetic Analysis Indicates High Evolutionary Conservation of the Killifish DNA Methylation Machinery

**FIGURE 1:**
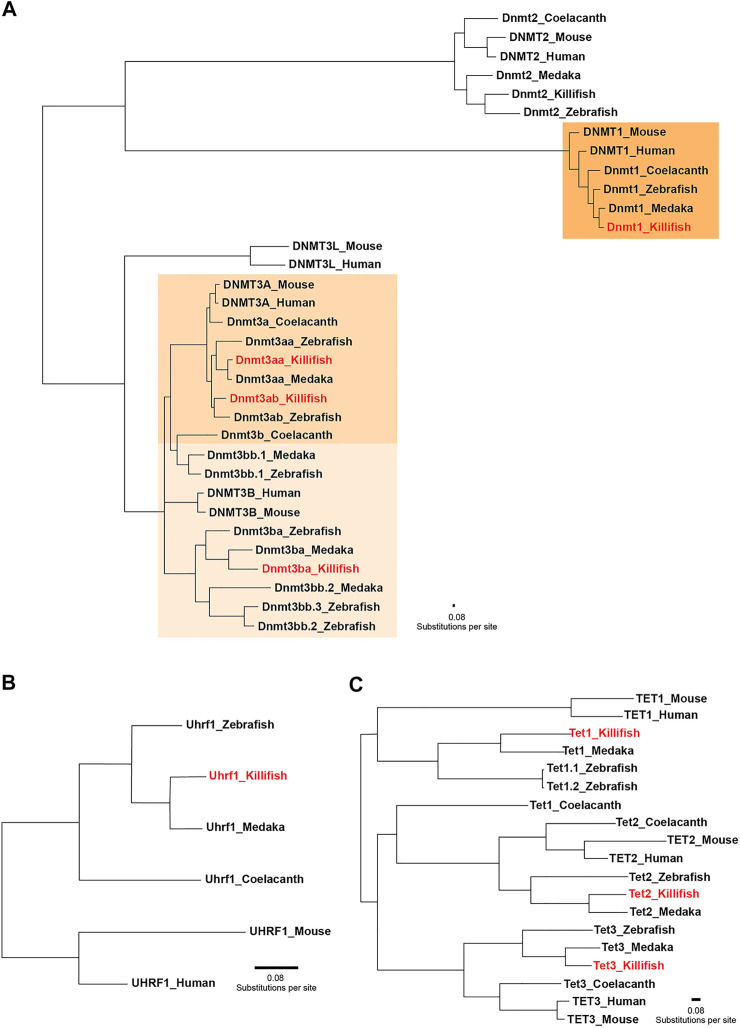
Phylogenetic relationships between key enzymes constituting the DNA methylation machinery. Phylogenetic tree of vertebrate DNA methyltransferases (DNMTs) **(A)** DNMT1 recruitment factor UHRF1 **(B)** and ten-eleven translocation methylcytosine dioxygenases (TETs) **(C)** computed by the Maximum-Likelihood algorithm. Full protein sequences obtained from the following species were used: human (*Homo sapiens*), mouse (*Mus musculus*), zebrafish (*Danio rerio*)*,* medaka (*Oryzias latipes*)*,* killifish (*Nothobranchius furzeri*), and coelacanth (*Latimeria chalumnae*)*.* Coelacanth represents a sarcopterygian fish illustrating the transition to a terrestrial life, creating modern tetrapods. The scale bar represents number of substitutions per site.

In order to assess the species-specific composition and evolutionary conservation of the killifish DNA methylation machinery, we surveyed both annotated genomes of *N. furzeri* (Reichwald et al., 2015; Valenzano et al., 2015). Data processing of the available databases retrieved five *dnmt* gene family orthologues corresponding to the mammalian *DNMTs* (*DNMT1, 2, 3A, and 3B*). Comparable to most vertebrates, the *N. furzeri* genome harbors one *dnmt1* orthologue and multiple copies (*dnmt3aa, dnmt3ab,* and *dnmt3ba*) of the *de novo* DNA methylation subfamily. Concordant with an expansion of the Dnmt3 subfamily in non-mammalian lineages, the zebrafish and the medaka genome contain six and four *dnmt3* genes, respectively (Goll and Halpern 2011). In addition to canonical cytosine-5 DNMTs, the killifish genome encodes one *dnmt2* orthologue. Although sharing a clear phylogenetic relationship with the DNMT family, DNMT2 does not exhibit DNA methylation activity and replaced its substrate DNA with RNA constituting a tRNA methyltransferase (Goll et al., 2006). A related search for the *DNMT1* recruitment factor *UHRF1* (Bostick et al., 2007; Sharif et al., 2007) and *TET* family members resulted in the identification of a single orthologue for *N. furzeri uhrf1, tet1, tet2,* and *tet3*. To examine the evolutionary relationships of killifish key enzymes constituting the DNA methylation machinery within the vertebrate system, we computed phylogenetic analysis on the protein level including human, mouse, coelacanth, zebrafish, medaka, and killifish. The rationale for including coelacanth in our analysis is its status as a living fossil, which transitioned to terrestrial life thereby founding modern tetrapods (Amemiya et al., 2013). A phylogenetic tree implementing the Maximum-Likelihood algorithm revealed that killifish Dnmts/recruitment factor Uhrf1 and Tets allocated unequivocally to the well-defined clades, displaying highest conservation with other teleost species, especially medaka (Figure 1). With the exception of Dnmt3ba, amino acid sequence similarity assessed through pairwise sequence comparison of full-length proteins showed a high degree of conservation of individual DNMT/UHRF1 and TET subfamilies. Scoring similarity between killifish and human were: Dnmt1: 74%, Uhrf1: 70%, Dnmt3aa: 73%, Dnmt3ab: 75%, Tet1: 39%, Tet2:49%, Tet3: 46% (Supplementary Table 2).

### Expression of Methylation Machinery Key Components Is Strongly Induced After Zygotic Gene Activation During Killifish Embryonic Development

**FIGURE 2:**
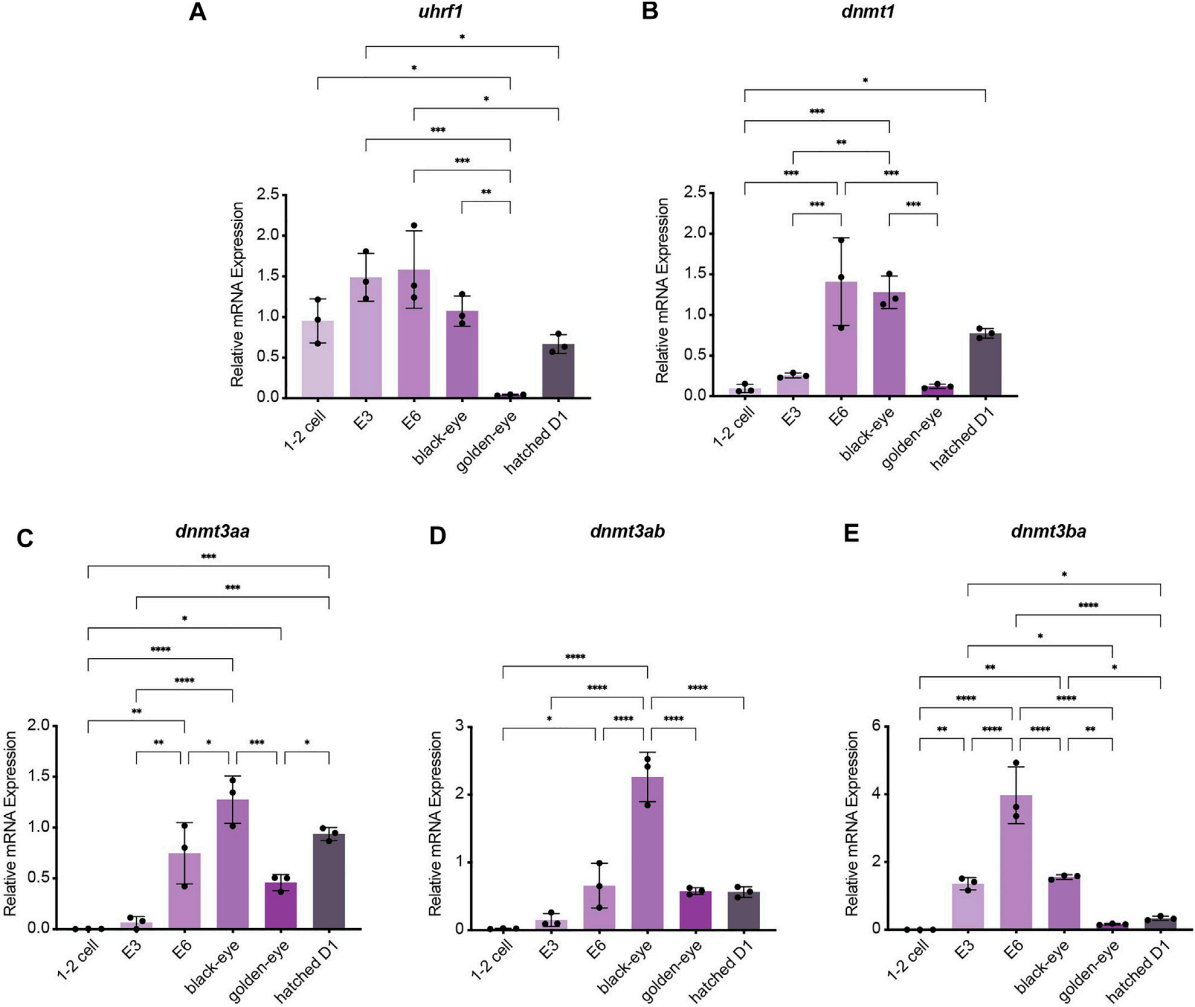
Gene expression profiling of killifish DNA methyltransferases and recruitment factors during embryogenesis and post hatching measured by Real-time qRT-PCR. DNMT1 recruitment factor *uhrf1* **(A)**, *dnmt1* **(B)**, *dnmt3aa* **(C)**, *dnmt3ab* **(D)**, *dnmt3ba* **(E)**. Individual *dnmt/uhrf1* expression was normalized to *tbp* housekeeping gene expression. For total RNA isolation the following numbers of embryos per biological replicate were sampled (1–2 cell: 200 eggs; E3 and E6: 100 eggs; black and golden eye: 50 eggs; hatched D1: 30 fish). All error bars represent SD of three biological replicates. Statistical significances were calculated with one-way ANOVA implementing Tukey’s multiple comparisons. All developmental stages (1–2 cell, E3, E6, black eye, golden eye, and newly hatched fish D1) where compared to each other and relative mRNA expression is depicted. ns: *p* > 0.05; *: *p* ≤ 0.05; **p ≤ 0.01; ***: *p* ≤ 0.001; ****: *p* ≤ 0.0001.

**FIGURE 3:**
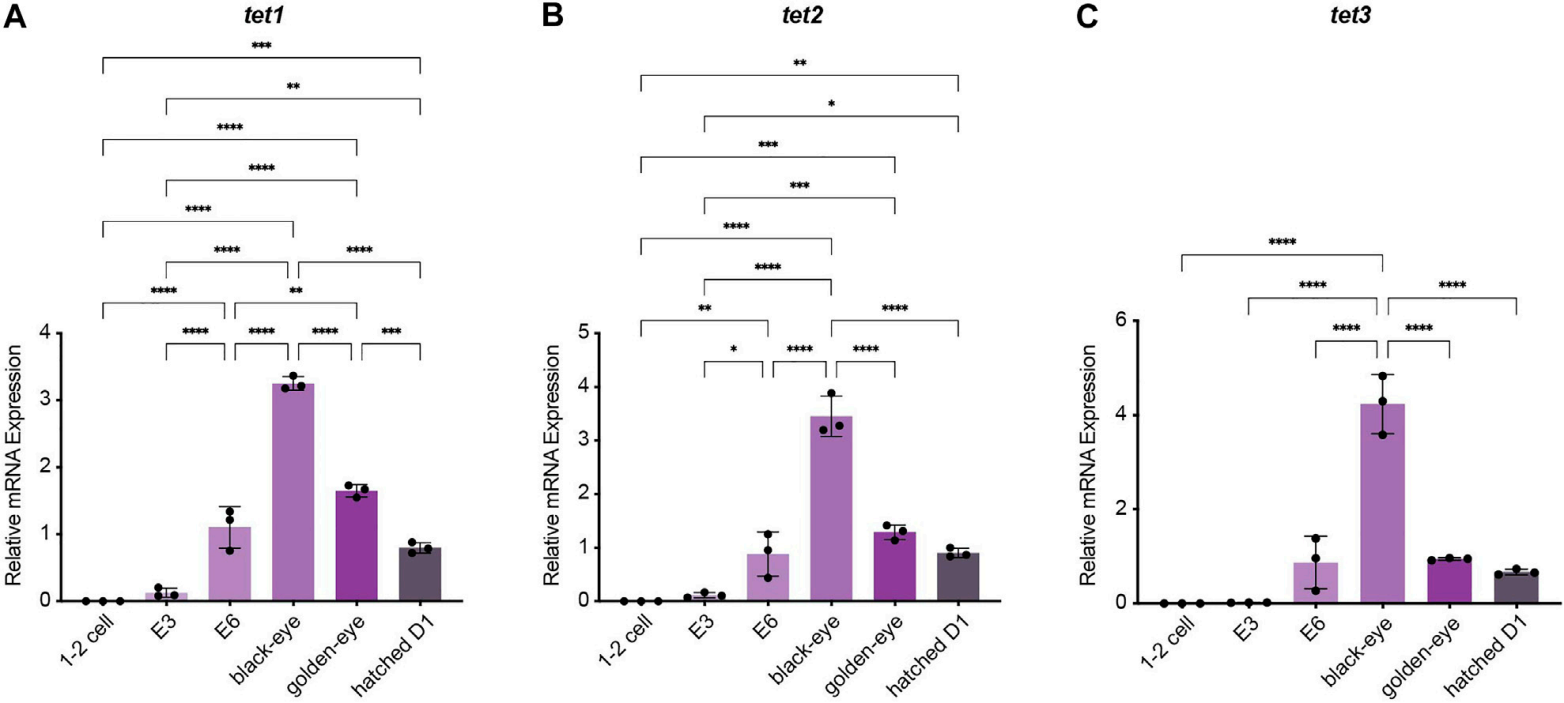
Expression analysis of the ten-eleven translocation methylcytosine dioxygenase family during embryogenesis and post hatching. Quantitative Real-time PCR of killifish *tet1* **(A)**, *tet2* **(B)**, *tet3* **(C)**. Individual *tet* expression was normalized to *tbp* housekeeping gene expression. For total RNA isolation the following numbers of embryos per biological replicate were sampled (1–2 cell: 200 eggs; E3 and E6: 100 eggs; black and golden eye: 50 eggs; hatched D1: 30 fish). All error bars represent SD of three biological replicates. Statistical significances were calculated with one-way ANOVA implementing Tukey’s multiple comparisons. All developmental stages (1–2 cell, E3, E6, black eye, golden eye, and newly hatched fish D1) where compared to each other and relative mRNA expression is depicted. ns: *p* > 0.05; *: *p* ≤ 0.05; **p ≤ 0.01; ***: *p* ≤ 0.001; ****: *p* ≤ 0.0001.

To investigate how methylation dynamics are regulated during embryogenesis and the very early life cycle of *N. furzeri,* we mapped expression of the most relevant regulatory genes setting the killifish methylome and hydroxymethylome. To cover early development from fertilization to post hatching we analyzed the following six time points by quantitative Real-time PCR: 1–2 cell stage, embryonic day 3 (E3) characterized by completed epiboly and the deep blastomeres are dispersed around the egg. Noteworthy, at this stage annual killifish embryos have the possibility to enter Diapause I (Naumann and Englert 2018; Dolfi et al., 2019). The next time point was embryonic day 6 (E6) of the phylotypic stage when embryos have reached mid-somitogenesis. The last two sampling points of embryonic development are termed “black eye” (organogenesis completed) and “golden eye” (embryo awaits a hatching stimulus). The final time point constituted newly hatched fish 24 h post hatching (D1). Expression of both factors involved in maintaining methylation patterns peaked at developmental day E6 with sustained transcript levels at the black eye stage followed by an extensive decline in golden eyes. After hatching, both enzymes *dnmt1* and *uhrf1* showed substantial re-expression (Figures 2A,B). Whereas *dnmt1* was strongly induced at E6 (about 15-fold) (Figure 2B), maternally deposited *uhrf1 mRNAs* were already detectable at significant levels at 1–2 cell stage before zygotic gene activation (ZGA) (Figure 2A). All *de novo* DNA methyltransferases showed a similar expression pattern characterized by a strong upregulation after ZGA, a subsequent increase to peak levels for *dnmt3ba* at E6 and *dnmt3aa* and *dnmt3ab* at the black eye stage. In contrast to *dnmt1/uhrf1,* members of the *dnmt3a* subfamily were abundantly expressed at the golden eye stage with stable transcript levels through the hatching process (Figures 2C–E). The three killifish orthologues of Tet enzymes involved in enzymatic DNA demethylation exhibited a uniform expression profile during embryonic development with *tet* mRNAs being virtually undetectable in the 1–2 cell stage. Tet expression was initiated after ZGA, displaying a strong induction as development proceeds peaking at the black eye stage followed by reduced but continuously high transcript levels at the golden eye stage and during hatching (Figures 3A–C).

### Components of the Killifish DNA Methylation Machinery Are Downregulated During the Aging Process

**FIGURE 4:**
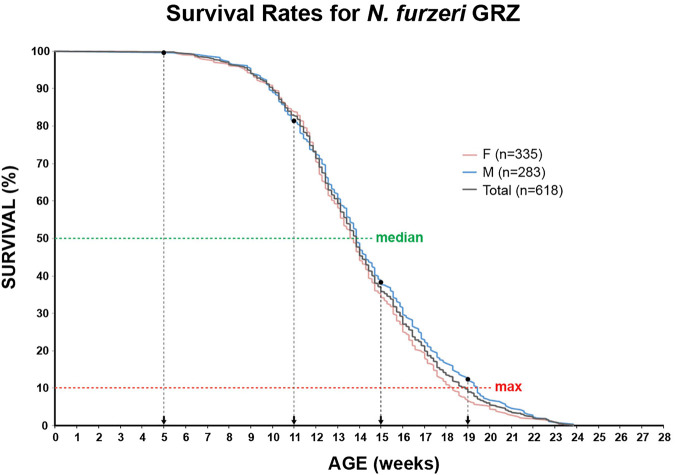
Survivorship curve of the *N. furzeri* GRZ strain. Kaplan Meier estimates incorporating age-dependent mortality data of ten consecutive hatches for female (*n* = 335) and male (*n* = 283) populations. Total number of animals *n* = 618. As fish reach their sexual maturity at 5 weeks, this age represents the experimental starting point. Median (50%) and maximum (90%) survival are illustrated as green and red horizontal, dashed lines, respectively. Selected time points for qRT-PCR analysis and global DNA methylation/hydroxymethylation quantification are indicated.

**FIGURE 5:**
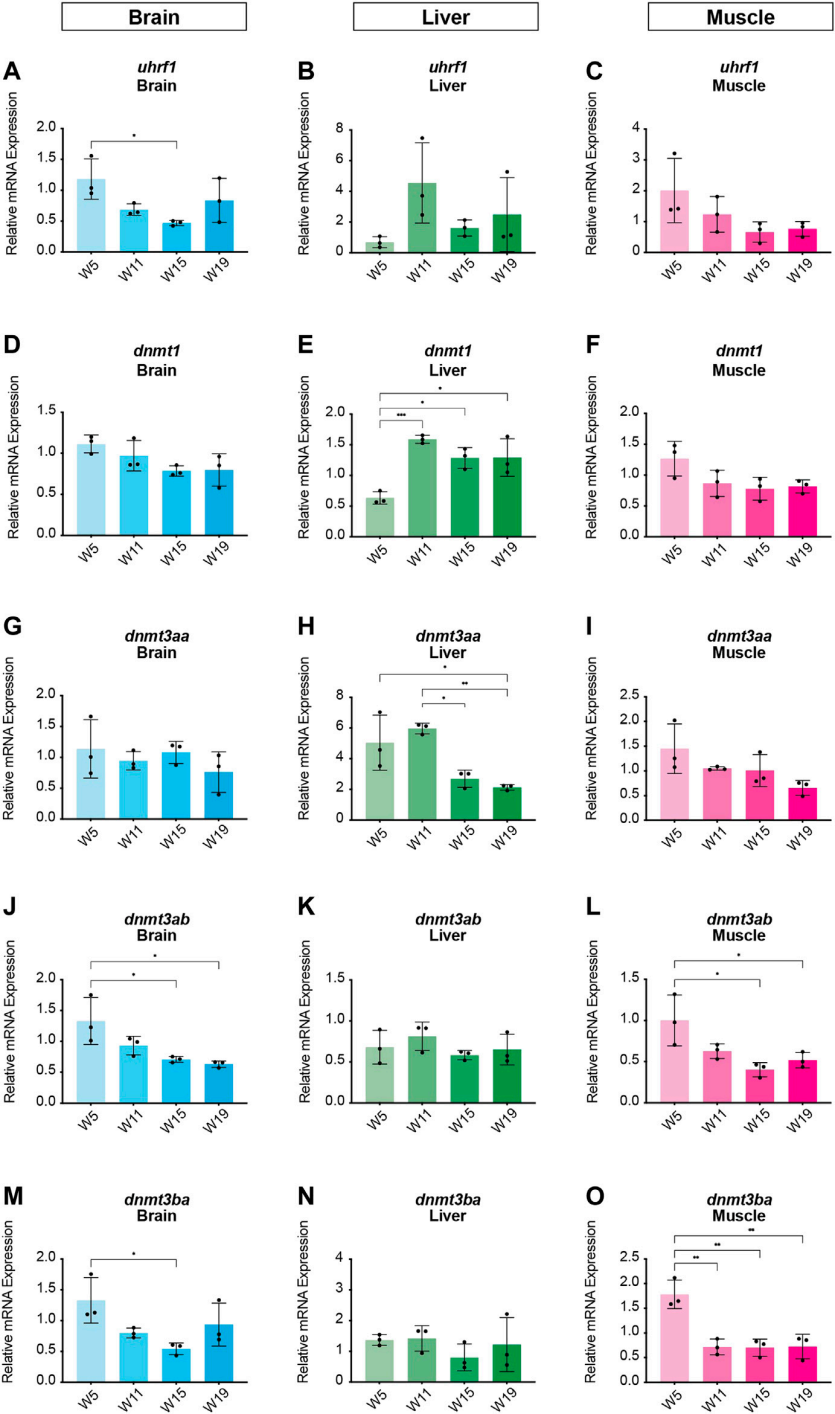
Age-dependent expression analysis of killifish DNA methyltransferases and recruitment factors. Quantitative Real-time PCR of killifish DNMT1 recruitment factor *uhrf1* **(A–C)**, *dnmt1* **(D–F)**, *dnmt3aa* **(G–I)**, *dnmt3ab* **(J–L)**, *dnmt3ba* **(M–O)** in brain, liver, and muscle at 5 (W5), 11 (W11), 15 (W15), and 19 (W19) weeks of age. Individual *dnmt/uhrf1* expression was normalized to *tbp* housekeeping gene expression. All error bars represent SD of three biological replicates. Statistical significances were calculated with one-way ANOVA using Tukey’s multiple comparisons comparing the means of all time points with each other. Relative mRNA expression is shown. ns: *p* > 0.05; *: *p* ≤ 0.05; **p ≤ 0.01; ***: *p* ≤ 0.001; ****: *p* ≤ 0.0001.

**FIGURE 6:**
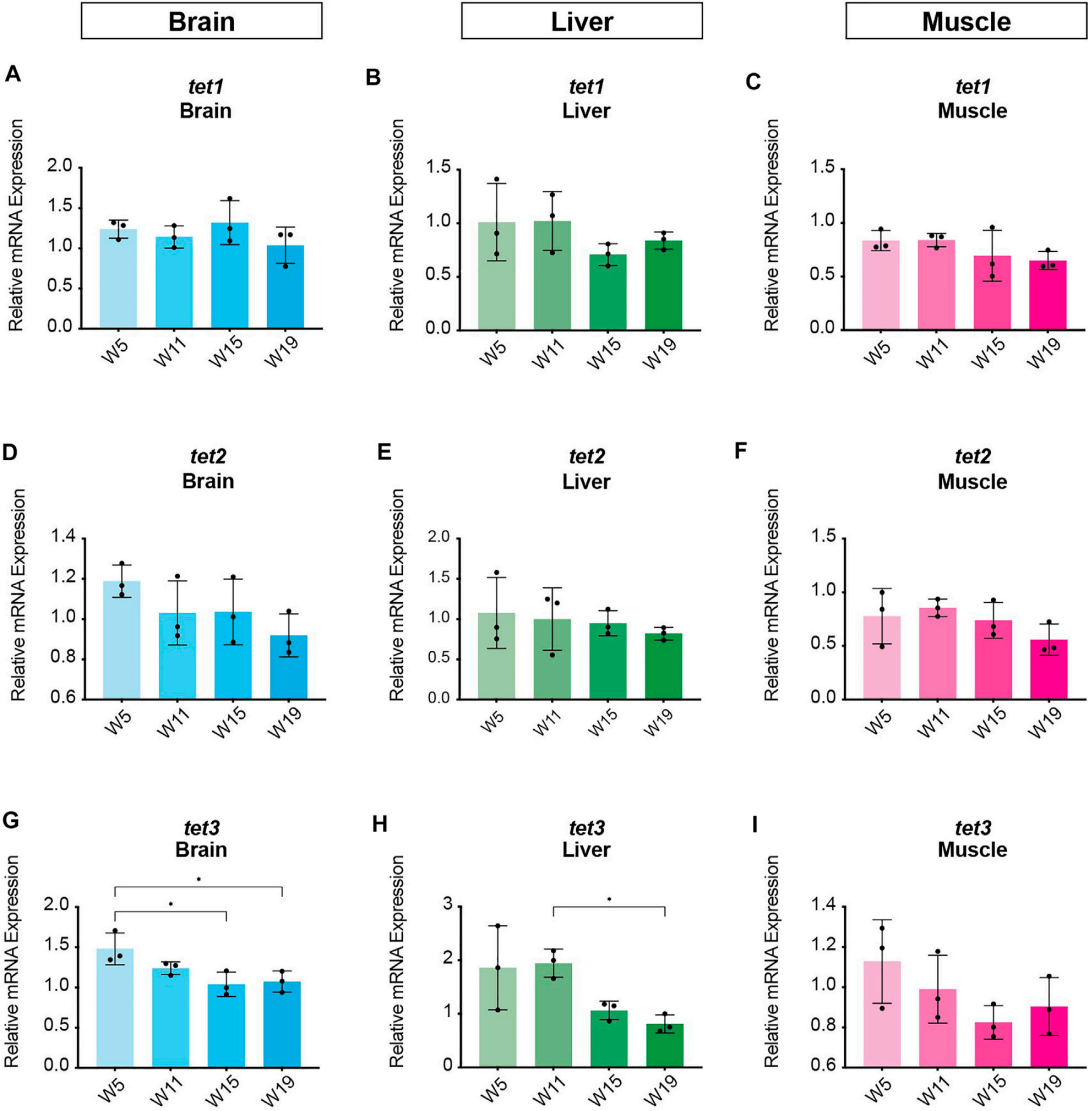
Age-dependent gene expression profiling of the killifish ten-eleven translocation methylcytosine dioxygenase family. Quantitative Real-time PCR of killifish *tet1* **(A–C)**, *tet2* **(D–F)**, *tet3* **(G–I)** in brain, liver and muscle at 5 (W5), 11 (W11), 15 (W15), and 19 (W19) weeks of age. Individual *tet* expression was normalized to *tbp* housekeeping gene expression. All error bars represent SD of three biological replicates. Statistical significances were calculated with one-way ANOVA using Tukey’s multiple comparisons comparing the means of all time points with each other. Relative mRNA expression is shown. ns: *p* > 0.05; *: *p* ≤ 0.05; **p ≤ 0.01; ***: *p* ≤ 0.001; ****: *p* ≤ 0.0001.

In order to identify potential changes in expression levels of the enzymatic machinery which sets, maintains, modifies and oxidates DNA methylation marks in *N. furzeri* as a function of age, we analyzed four different time points in three functionally different tissues including brain, liver and muscle. For determination of relevant time points representing the different aging phases in the adult life cycle, we determined the survival of the *N. furzeri* GRZ strain (Figure 4). Noteworthy, the survival rates are in agreement with datasets described by other *N. furzeri* facilities (Hartmann et al., 2009; Smith et al., 2017). As a starting time point, we used 5 weeks post-hatching (W5) when the fish reaches sexual maturity. Eleven weeks of age (W11) corresponds to approximately 80% survivorship. At the third time point, week 15 (W15), survivorship has already decreased to about 40% and the fish start showing first aging-related characteristics. The last time point in our analysis defines week 19 (W19), correlating with approximately 10% survivorship. At this age, most fish display a drastic decline in performance featuring aging-related characteristics, such as spine curvature malformations, reduced muscle mass and emaciation. In order to survey expression levels of DNA methylation machinery enzymes, we extracted total RNA from dissected brain, liver and muscle tissues followed by quantitative Real-time PCR (qRT-PCR). DNA methylation maintenance factors *dnmt1* and *uhrf1* displayed a predominantly age-dependent decrease in transcriptional activity in all analyzed tissues (Figures 5A–F), most prominently observed in skeletal muscle (Figures 5C,F). In contrast to a small but gradual downregulation in brain and muscle, expression of *dnmt1* in the liver was characterized by a significant induction after week 5, reaching peak levels at week 11 followed by a minor decrease at advanced age (Figure 5E). Although not statistically significant in all tissues, *de novo* DNA methyltransferases tended towards declining transcriptional activity with age (Figure 5G–O). Similar to *dnmt1/uhrf1* expression patterns, age-dependent downregulation of the *dnmt3* subfamily was most pronounced in muscle (Figure 5I, L, O). Expression profiling of the three members of the Tet family of methylcytosine dioxygenases catalyzing iterative oxidation of 5mC revealed a general trend of decreasing transcript levels with a significant reduction of *tet3* mRNAs in brain and liver (Figure 6). Collectively, all analyzed components constituting the killifish DNA methylation machinery predominantly revealed a negative correlation with age in multiple, functionally different tissues.

### The Killifish Genome Shows Tissue Specific Global DNA Hypomethylation as a Function of Age

**FIGURE 7:**
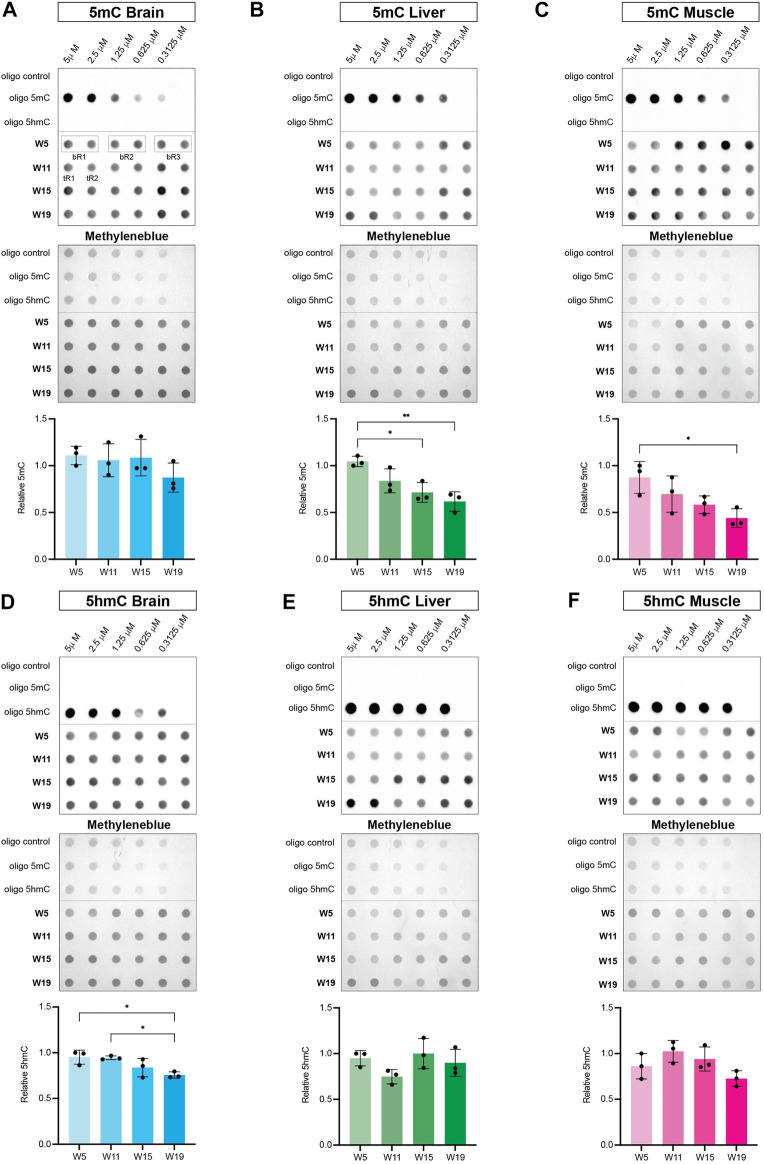
Dynamics of global DNA methylation **(A–C)** and DNA hydroxymethylation **(D–F)** in brain, liver, and muscle during the killifish aging process. Dot blot of modified control oligonucleotides in a 1:2 dilution series starting at 5 μM (unmethylated: oligo control, 5-methylated CpG: oligo 5mC and 5-hydroxymethylated CpG: oligo 5hmC) **(upper panel)** and genomic DNA isolated from brain, liver and muscle at 5 (W5), 11 (W11), 15 (W15), and 19 (W19) weeks of age **(lower panel)** spotted in biological triplicates (bR1, 2, 3) composed of two technical replicates (tR1, 2). The membrane was probed with primary antibodies against 5mC and 5hmC followed by incubation with secondary antibodies (anti-rabbit/anti-mouse IgG-HRP). Levels of 5mC and 5hmC were normalized to the respective methylene blue signal intensities and calculated as mean of three biological triplicates per time point. Statistical significances were calculated with one-way ANOVA and multiple comparisons were performed by comparing W11, W15 and W19 to W5 setting one biological replicate to 1. ns: *p* > 0.05; *: *p* ≤ 0.05; **p ≤ 0.01; ***: *p* ≤ 0.001; ****: *p* ≤ 0.0001.

To correlate age-dependent expression profiles of the killifish DNA methylation machinery with epigenetic changes in the methylome and hydroxymethylome, we performed dot blot assays with specific antibodies against 5mC and 5hmC. Of note, modified control oligonucleotides (unmethylated, 5-methylated CpG, and 5-hydroxymethylated CpG) were used to evaluate specificity of antibodies. Analysis of global DNA methylation showed a significant negative correlation of 5mC with age in liver and muscle tissue. Levels of 5mC did not show a particular age-dependent regulation in brain, whereas global loss of methylation was most pronounced in skeletal muscle (Figures 7A–C). Notably, global hypomethylation in muscle strongly coincided with a decline in transcript levels of both, maintenance and *de novo* DNA methyltransferases with aging (Figures 5F, I, L, O). Regarding global 5hmC levels, a significant age-associated downregulation was only observed for brain (Figure 7D) concurring with a substantial reduction in *tet3* transcriptional activity (Figure 6G). In contrast, DNA hydroxymethylation in liver and muscle showed stable 5hmC levels throughout the aging process (Figures 7E,F). Overall, age-dependent transcriptional profiles of the key factors defining the killifish DNA methylation machinery exhibited a strong positive correlation with chronological alterations of global DNA methylation and hydroxymethylation patterns.

## Discussion

Mining of the *N. furzeri* genome databases (Reichwald et al., 2015; Valenzano et al., 2015) produced evidence that killifish encodes all the key components defining the DNA methylation machinery. Similar to most vertebrates, we identified a single orthologue of mammalian maintenance enzymes DNMT1, its recruitment factor UHRF1 and the three TET family members involved in iterative oxidation of 5mC. Additionally, the killifish genome includes a single homologue of human DNMT2. DNMT2 proteins represent the most widely conserved family of DNA methyltransferase paralogues in eukaryotes. However, DNMT2 lacks robust DNA methylation activity *in vitro* and *in vivo* but instead switched its substrate specificity from DNA to RNA, methylating a small set of tRNAs (Goll et al., 2006). Regarding *de novo* DNA methyltransferases of the DNMT3 family, killifish possess two orthologues of DNMT3A and a single homologue of DNMT3B. The presence of two DNMT3A orthologues appears to be conserved in many fish species, as two genes specifying Dnmt3a-like proteins are also present in the genomes of zebrafish, *Tetraodon*, fugu, and stickleback (Goll and Halpern 2011). Copy numbers of the DNMT3B subfamily showed the greatest variability with one, three and four copies for killifish, medaka and zebrafish, respectively. Not surprisingly, DNMT3L orthologues are absent in killifish. The mammalian-specific DNMT3L is a truncated DNMT3 variant. Despite lacking catalytic activity, DNMT3L is essential for methylation at monoallelically expressed imprinted genes in the maternal genome (Bourc’his et al., 2001). Unlike mammals, teleost species do not require genomic imprinting (Goll and Halpern 2011). However, DNMT3L is also required for the methylation of transposable elements in the paternal mouse genome (Bourc’his and Bestor 2004), and methylation has been detected at transposable elements in zebrafish (Feng et al., 2010). It will be interesting to determine whether *de novo* methylation of transposable elements occurs in the germline of *N. furzeri* and, if so, whether any of the killifish specific Dnmt3 orthologues compensate for DNMT3L in this process. In summary, killifish uses essentially the same enzymatic machinery as mouse and human to write, read and erase DNA methylation marks.

The fundamental variations of early embryonic development between amniotes and mammals are reflected by remarkable differences in their usage of the DNA methylation machinery. In mammalian development, two waves of DNA methylation reprogramming occur: 1) before zygotic maternal and paternal pronuclei fusion and 2) in primordial germ cell (PGCs) specification. Each reprogramming window is characterized by global DNA demethylation resetting the epigenome to provide a scaffold for the launch of novel epigenetic marks to either maintain the totipotent state and consequent lineage decisions or to establish germ cell identity in PGCs (Seisenberger et al., 2013). Confounding observations have been reported with regard to DNA methylome reprogramming after fertilization and PGC development in anamniote vertebrates. A large body of elegant work has demonstrated the absence of genome-wide DNA methylome reprogramming in zebrafish and *Xenopus* embryos (Macleod et al., 1999; Veenstra and Wolffe 2001; Hontelez et al., 2015; Skvortsova et al., 2019). In contrast to mammalian DNA methylation erasure and re-establishment, zebrafish embryos achieve totipotency by a maternal to paternal 5mC reconfiguration prior to zygotic genome activation (Jiang et al., 2013; Potok et al., 2013). In stark contrast to these findings, two very recent studies have reported a significantly different scenario regarding DNA methylation dynamics after fertilization (Wang and Bhandari 2019) and epigenetic reprogramming of primordial germ cells (Wang and Bhandari 2020) in the teleost model medaka. Specifically, the authors observed active DNA demethylation within the first cell cycle which coincided with detectable Tet expression mirroring the mammalian mechanism of global DNA methylation erasure (Wang and Bhandari 2019). Regarding these surprising mechanistic discrepancies in DNA methylation reprogramming between the two teleost models zebrafish and medaka, we profiled the expression of the killifish DNA methylation machinery from the 1–2 cell stage until post hatching. Overall, all DNA methyltransferases and Tet enzymes showed a strong upregulation after ZGA with peak levels during the phylotypic stage, the most conserved period of vertebrate embryogenesis, characterized by pan-vertebrate gene-regulatory conformities (Figures 2, 3). In particular, transcripts of the three Tet family members were virtually undetectable in the 1–2 cell stage (Figure 3). This is in accordance with previous studies in zebrafish reporting extremely low Tet expression in pre-gastrula embryos and no observable 5hmC enrichment before the onset of organogenesis (Almeida et al., 2012; Potok et al., 2013; Bogdanović et al., 2016). Collectively, our expression data in killifish favor a DNA methylation remodeling mechanism as described in zebrafish. More extensive mechanistic studies will be necessary in resolving questions related to the conservation of 5mC reprogramming during the early teleost life cycle.

It is well established that aging is linked to extensive changes in the DNA methylation landscape throughout life (Ciccarone et al., 2018). Several lines of evidence indicate that the observed epigenetic alterations during the aging process could be caused by modified expression levels of the DNA methylation machinery (Xiao et al., 2008; Li et al., 2010; Qian and Xu 2014; Ciccarone et al., 2016). To address this issue in *N. furzeri*, we analyzed age-associated transcriptional regulation of DNA methyltransferases and Tet methylcytosine dioxygenases in functionally different tissues including brain, liver and muscle. We observed tissue-specific expression profiles for all analyzed enzymes displaying a common tendency towards decreased transcriptional activity with age (Figures 5, 6). Downregulation of maintenance and *de novo* methyltransferases was most pronounced in liver and skeletal muscle. Notably, on an organismic level, aging in *N. furzeri* is reflected by reduced muscle mass paralleled by declining locomotor activity (Platzer and Englert 2016). Unexpectedly, given the killifish’s short lifespan, frequent incidences of age-dependent liver neoplasias in *N. furzeri* have been reported (Di Cicco et al., 2011). It is tempting to speculate that transcriptional deregulation of methyltransferases resulting in altered DNA methylation patterns might play a role in the liver-specific emergence of tumors. Particularly, with regard to *dntm3* isoform usage we observed a significant downregulation of *de novo* methyltransferase *dnmt3aa* in liver, whereas in brain and muscle transcription of *dnmt3ab* and *dnmt3ba* was decreased. These data suggest a diverse assignment of the DNA *de novo* methylation members in a tissue-specific manner. In brain, we detected significant downregulation of the maintenance methyltransferase *dnmt3ab* whereas the other *dnmts* remained relatively stable throughout aging. A similar age-dependent decline of the *Dnmt3a2*-isoform was reported in the mouse brain where re-expression in the hippocampus was able to restore cognitive abilities of aged animals (Oliveira et al., 2012).

Finally, we correlated age-dependent expression profiles of the killifish DNA methylation machinery with epigenetic changes in the methylome and hydroxymethylome (Figure 7). Intriguingly, age-dependent downregulation of DNA methyltransferases in liver and muscle coincided with significant global hypomethylation in both tissues whereas the brain revealed relatively stable 5mC levels upon aging. In mammals, global 5mC levels in postmitotic neurons similarly appear stable throughout their lifetime (Lister et al., 2013). This consistent DNA methylation is suggested to define cellular identity and might be a direct consequence of prevented passive loss of DNA methylation through cell division. As liver and muscle cells undergo continuous cell cycles throughout adult life, the global loss of DNA methylation might be more apparent in these tissues. Additionally, CH methylation (where H is C, A or T) becomes particularly abundant after synapse formation in mammalian brain and remains constant throughout life (Lister et al., 2013). As our dot blots using antibodies against 5mC do not differentiate between mCG and mCH, these dynamic changes might not be reflected in our experiments. However, the levels of non-CG methylation in *N. furzeri* have not been determined to date and we can only speculate that CH in fish is comparably methylated reflecting the mammalian system. Changes in global DNA methylation have previously been linked to cognitive regression and aging. It is highly possible that these methylation changes occur on specific DNA elements such as enhancers or gene bodies rather than on a global scale (Li et al., 2019). As our dot blot analysis only measures global 5mC abundance, it will be important to perform whole-genome bisulfite analyses of the *N. furzeri* methylome to stratify genomic regions affected by dynamic DNA methylation changes inflicted by age. 5hmC is a relatively novel epigenetic mark that is most abundant in mammalian postmitotic neurons and embryonic stem cells (Kriaucionis and Heintz 2009; Tahiliani et al., 2009). To identify 5hmC signatures in *N. furzeri*, we performed dot blots with an 5hmC antibody (Figure 7). Comparable to the mammalian system, we detected pronounced 5hmC levels in the brain and to a lesser extend in liver and muscle. In killifish muscle and liver, 5hmC levels remained constant but showed a modest decrease in brain. This is in contrast to a recent study which reported upregulation of 5hmC in at least some regions of aged mouse brains suggesting that 5hmC has important functions in neurodevelopment and aging (Szulwach et al., 2011). After successful identification of 5hmC in killifish, future next generation sequencing (NGS) experiments will be essential to compare these signatures to mammalian 5hmC patterns and identify similar or divergent functions of 5hmC in *N. furzeri*.

During the past decades, extensive effort has been made to identify aging biomarkers to predict chronological and biological age across different species and various cell types. Recent advances in epigenomics have resulted in novel prediction tools that allow precise age estimation based on DNA methylation data outperforming all previously proposed biomarkers including telomere attrition, alterations in gene expression and metabolite concentrations (Jylhävä et al.,). Although these epigenetic aging clocks were initially described in pioneering human studies (Bocklandt et al., 2011; Hannum et al., 2013; Horvath 2013; Weidner et al., 2014) they have since been replicated in mice (Petkovich et al., 2017; Stubbs et al., 2017; Wang et al., 2017) and to some degree in dogs, wolves (Thompson et al., 2017), and humpback whales (Polanowski et al., 2014). To the best of our knowledge no DNA methylation based epigenetic clocks have been reported for non-mammalian vertebrates. Collectively, our data showing that the killifish DNA methylation machinery is highly conserved and that changes in its expression levels correlate with altered, tissue-specific DNA methylation patterns during the aging process point towards the exciting possibility to develop an analogous epigenetic aging clock in killifish. Such a biomarker will constitute a useful instrument to examine and evaluate geroprotective interventions in the new vertebrate model system *N. furzeri.*

## Data Availability

The original contributions presented in the study are included in the article/Supplementary Material, further inquiries can be directed to the corresponding author.
