# Supplementary material for: Analysis of Methylation Dynamics Reveals a Tissue-Specific, Age-Dependent Decline in 5-Methylcytosine Within the Genome of the Vertebrate Aging Model Nothobranchius furzeri
Source: Front Mol Biosci. 2021 Jun 16;8:627143. doi: 10.3389/fmolb.2021.627143 (PMC8242171; doi:10.3389/fmolb.2021.627143)
Supplement: Supplementary file 1 [file DataSheet1.pdf]

SUPPLEMENTARY MATERIAL

Supplementary Table 1

Accession Numbers		
**DNMTs**		
**Species**	**Gene / Protein**	**ENSEMBL/NFINgb Accession**
H. sapiens	DNMT1	ENSP00000352516
H. sapiens	DNMT2	ENSP00000367030
H. sapiens	DNMT3A	ENSP00000264709
H. sapiens	DNMT3B	ENSP00000328547
H. sapiens	DNMT3L	ENSP00000270172
M.musculus	DNMT1	ENSMUSP00000136669
M.musculus	DNMT2	ENSMUSP00000114572
M.musculus	DNMT3A	ENSMUSP00000020991
M.musculus	DNMT3B	ENSMUSP00000051830
M.musculus	DNMT3L	ENSMUSP00000121562
Latimeria chalumnae	Dnmt1	ENSLACP00000012502
Latimeria chalumnae	Dnmt2	ENSLACP00000015770
Latimeria chalumnae	Dnmt3a	ENSLACP00000014690
Latimeria chalumnae	Dnmt3b	ENSLACP00000009510
D. rerio	Dnmt1	ENSDARP00000108456
D. rerio	Dnmt2	ENSDARP00000048632
D. rerio	Dnmt3aa	ENSDARP00000091093
D. rerio	Dnmt3bb	ENSDARP00000126446
D. rerio	Dnmt3ba	ENSDARP00000108732
D. rerio	Dnmt3bb.1	ENSDARP00000053417
D. rerio	Dnmt3bb.2	ENSDARP00000074994
D. rerio	Dnmt3bb.3	ENSDARP00000118597
O. latipes	Dnmt1	ENSORLP00000007019
O. latipes	Dnmt2	ENSORLP00000024161
O. latipes	Dnmt3aa	ENSORLP00000022087
O. latipes	Dnmt3ba	ENSORLP00000018521
O. latipes	Dnmt3bb.1	ENSORLP00000025108
O. latipes	Dnmt3bb.2	ENSORLP00000027779
N. furzeri	Dnmt1	Nfu_g_1_007742
N. furzeri	Dnmt2	Nfu_g_1_013450
N. furzeri	Dnmt3aa	Nfu_g_1_019101
N. furzeri	Dnmt3ab	Nfu_g_1_021728
N. furzeri	Dnmt3ab	Nfu_g_1_001897
**TETs**		
**Species**	**Gene / Protein**	**ENSEMBL/NFINgb Accession**
H. sapiens	TET1	ENSP00000362748
H. sapiens	TET2	ENSP00000369351
H. sapiens	TET3	ENSP00000307803
M.musculus	TET1	ENSMUSP00000059527
M.musculus	TET2	ENSMUSP00000096203

M.musculus	TET3	ENSMUSP00000139630
Latimeria chalumnae	Tet1	ENSLACP00000016889
Latimeria chalumnae	Tet2	ENSLACP00000008576
Latimeria chalumnae	Tet3	ENSLACP00000009535
D. rerio	Tet1.1	ENSDARP00000104252
D. rerio	Tet1.2	ENSDARP00000143362
D. rerio	Tet2	ENSDARP00000101295
D. rerio	Tet3	ENSDARP00000145927
O. latipes	Tet1	ENSORLP00000029166
O. latipes	Tet2	ENSORLP00000032570
O. latipes	Tet3	ENSORLP00000041944
N. furzeri	Tet1	Nfu_g_1_014447
N. furzeri	Tet2	Nfu_g_1_018209
N. furzeri	Tet3	Nfu_g_1_009247
**UHRF1**		
**Species**	**Gene / Protein**	**ENSEMBL/NFINgb Accession**
H. sapiens	UHRF1	ENSP00000484739
M.musculus	UHRF1	ENSMUSP00000001258
Latimeria chalumnae	Uhrf1	ENSLACP00000016084
D. rerio	Uhrf1	ENSDARP00000135054
O. latipes	Uhrf1	ENSORLP00000008427
N. furzeri	Uhrf1	Nfu_g_1_002580

Supplementary Table 2

**A. DNMT1 Pairwise protein sequence comparison (percentage similarity)**						
	**Human DNMT1**	**Mouse DNMT1**	**Coelacanth Dnmt1**	**Zebrafish Dnmt1**	**Medaka Dnmt1**	**Killifish Dnmt1**
**Human DNMT1**		75.74	84.41	74.24	73.52	74.16
**Mouse DNMT1**	75.74		82.05	74.12	74.50	75.40
**Coelacanth Dnmt1**	84.41	82.05		86.21	85.78	85.99
**Zebrafish Dnmt1**	74.24	74.12	86.21		83.34	83.91
**Medaka Dnmt1**	73.52	74.50	85.78	83.34		88.21
**Killifish Dnmt1**	74.16	75.40	85.99	83.91	88.21	

**B. UHRF1 Pairwise protein sequence comparison (percentage similarity)**						
	**Human UHRF1**	**Mouse UHRF1**	**Coelacanth Uhrf1**	**Zebrafish Uhrf1**	**Medaka Uhrf1**	**Killifish Uhrf1**
**Human UHRF1**		86.66	79.45	82.63	80.67	69.45
**Mouse UHRF1**	86.66		73.68	78.14	76.32	65.41
**Coelacanth Uhrf1**	79.45	73.68		83.74	80.87	69.04
**Zebrafish Uhrf1**	82.63	78.14	83.74		89.41	76.84
**Medaka Uhrf1**	80.67	76.32	80.87	89.41		80.62
**Killifish Uhrf1**	69.45	65.41	69.04	76.84	80.62	

C. DNMT3A Pairwise protein sequence comparison (percentage similarity)

	Human DNMT3A	Mouse DNMT3A	Coelacanth Dnmt3a	Zebrafish Dnmt3aa	Zebrafish Dnmt3ab	Medaka Dnmt3aa	Killifish Dnmt3aa	Killifish Dnmt3ab
**Human DNMT3A**		97.48	86.05	70.69	70.25	84.03	72.89	74.62
**Mouse DNMT3A**	97.48		86.63	71.00	71.06	84.40	72.97	75.90
**Coelacanth Dnmt3a**	86.05	86.63		72.18	75.00	79.39	77.65	78.76
**Zebrafish Dnmt3aa**	70.69	71.00	72.18		66.42	72.51	70.14	68.98
**Zebrafish Dnmt3ab**	70.25	71.06	75.00	66.42		75.35	65.31	76.33
**Medaka Dnmt3aa**	84.03	84.40	79.39	72.51	75.35		91.93	78.38
**Killifish Dnmt3aa**	72.89	72.97	77.65	70.14	65.31	91.93		67.50
**Killifish Dnmt3ab**	74.62	75.90	78.76	68.98	76.33	78.38	67.50	

E. DNMT3B Pairwise protein sequence comparison (percentage similarity)

	Human DNMT3B	Mouse DNMT3B	Coelacanth Dnmt3B	Zebrafish Dnmt3ba	Zebrafish Dnmt3bb.1	Zebrafish Dnmt3bb.2	Zebrafish Dnmt3bb.3	Medaka Dnmt3ba	Medaka Dnmt3bb1	Medaka Dnmt3bb2	Killifish Dnmt3ba
**Human DNMT3B**		86.45	68.65	38.12	64.44	38.39	39.46	38.76	63.75	36.57	16.62
**Mouse DNMT3B**	86.45		67.87	37.69	64.21	38.71	39.09	37.68	63.91	36.87	16.59
**Coelacanth Dnmt3b**	68.65	67.87		56.92	72.11	52.97	54.20	55.86	73.36	47.42	23.33
**Zebrafish Dnmt3ba**	38.12	37.69	56.92		38.15	52.94	51.14	61.86	38.01	44.90	44.42
**Zebrafish Dnmt3bb.1**	64.44	64.21	72.11	38.15		39.37	39.38	39.65	77.91	35.38	17.36
**Zebrafish Dnmt3bb.2**	38.39	38.71	52.97	52.94	39.37		81.89	51.38	38.62	48.18	31.55
**Zebrafish Dnmt3bb.3**	39.46	39.09	54.20	51.14	39.38	81.89		50.57	39.40	48.61	29.33
**Medaka Dnmt3ba**	38.76	37.68	55.86	61.86	39.65	51.38	50.57		39.61	44.03	48.29
**Medaka Dnmt3bb1**	63.75	63.91	73.36	38.01	77.91	38.62	39.40	39.61		34.52	17.03
**Medaka Dnmt3bb2**	36.57	36.87	47.42	44.90	35.38	48.18	48.61	44.03	34.52		24.80
**Killifish Dnmt3ba**	16.62	16.59	23.33	44.42	17.36	31.55	29.33	48.29	17.03	24.80	

F. TET1 Pairwise protein sequence comparison (percentage similarity)

	Human TET1	Mouse TET1	Coelacanth TET1	Zebrafish Tet1.1	Zebrafish Tet1.2	Medaka Tet1	Killifish Tet1
**Human TET1**		62.84	35.25	39.55	39.44	39.03	38.63
**Mouse TET1**	62.84		32.21	39.56	39.53	38.39	37.32
**Coelacanth Tet1**	35.25	32.21		38.87	38.81	37.67	36.27
**Zebrafish Tet1.1**	39.55	39.56	38.87		99.89	48.91	47.93
**Zebrafish Tet1.2**	39.44	39.53	38.81	99.89		48.97	48.12
**Medaka Tet1**	39.03	38.39	37.67	47.93	48.12		60.55
**Killifish Tet1**	38.63	37.32	36.27	47.93	48.12	60.55	

G. TET2 Pairwise protein sequence comparison (percentage similarity)						
	**Human TET2**	**Mouse TET2**	**Coelacanth Tet2**	**Zebrafish Tet2**	**Medaka Tet2**	**Killifish Tet2**
**Human TET2**		65.12	56.58	49.15	49.73	48.83
**Mouse TET2**	65.58		54.31	44.30	44.13	44.88
**Coelacanth Tet2**	65.12	54.31		51.03	48.68	49.21
**Zebrafish Tet2**	49.15	44.30	51.03		55.28	54.23
**Medaka Tet2**	49.73	44.13	48.68	55.28		73.38
**Killifish Tet2**	48.83	44.88	49.21	54.23	73.38	

H. TET3 Pairwise protein sequence comparison (percentage similarity)						
	**Human TET3**	**Mouse TET3**	**Coelacanth Tet3**	**Zebrafish Tet3**	**Medaka Tet3**	**Killifish Tet3**
**Human TET3**		88.84	62.86	46.22	46.93	46.03
**Mouse TET3**	88.84		60.50	47.48	45.77	46.12
**Coelacanth Tet3**	62.86	60.50		46.00	47.96	46.39
**Zebrafish Tet3**	46.22	47.48	46.00		58.06	58.01
**Medaka Tet3**	46.93	45.77	47.96	58.06		76.36
**Killifish Tet3**	46.03	46.12	46.39	58.01	76.36	

Supplementary Table 3

Primer Name	Sequence (5’to 3’)
Dnmt1_RT-F<br>Dnmt1_RT-R	AGCAGCCCATCATTGTGAC<br>TGCTGAATCTGACCTGAGAAG
Uhrf1_RT-F<br>Uhrf1 RT-R	ACGCTGTCAAGAGCAAGGAC<br>GCGTCCACCAGCTCATTTAT
Dnmt3aa_RT-F<br>Dnmt3aa_RT-R	CTCAGCCGCTGGAAGATG<br>AACAGGCTCTGGTGTCACG
Dnmt3ab_RT-F<br>Dnmt3ab_RT-R	TGCCCAGAGTAACCTTCCAG<br>GTTGAAGGCTGTGGGTGTTT
Dnmt3ba_RT-F<br>Dnmt3ba_RT-R	TCCACGATCCTCCCTTGATA<br>GCCTGCAGTCATCCTCACTT
Tet1_RT-F<br>Tet1_RT-R	ACCAGATATGTAAGCTGAGGAAGTG<br>TGAGATTGTAAATGAGGTATGTGGTCT
Tet2_RT-F<br>Tet2_RT-R	CCATGCCCACAGAGACCTT<br>CTTGGATGGCCCCTGACTT
Tet3_RT-F<br>Tet3_RT-R	TTCTGGAATCACTGCTTGTATGG<br>GCCTCTTCACTACCAAACTCATC
*Tbp_RT-F<br>*Tbp_RT-R	CGGTTGGAGGGTTTAGTCCT<br>GCAAGACGATTCTGGGTTTG

*(Baumgart et al., 2014)

Baumgart, M., Groth, M., Priebe, S., Savino, A., Testa, G., Dix, A., Ripa, R., Spallotta, F., Gaetano, C., Ori, M., Terzibasi Tozzini, E., Guthke, R., Platzer, M., Cellerino, A., 2014. RNA-seq of the aging brain in the short-lived fish N. furzeri - conserved pathways and novel genes associated with neurogenesis. Aging Cell 13, 965–974. doi:10.1111/acel.12257

Supplementary Table 4

4A. qRT-PCR analysis (embryogenesis to post hatching) – TBP normalized mRNA expression						
	**1-2 cell**	**E3**	**E6**	**black-eye**	**golden-eye**	**hatched D1**
**DNMT1**	0,15391215	0,25030907	1,92129636	1,13177252	0,11985651	0,77520191
	0,0611077	0,28796221	1,4660758	1,50662591	0,15021883	0,83469057
	0,0701059	0,22937155	0,84313098	1,20096014	0,09842693	0,71500829
**DNMT3AA**	0,00087059	0,08084177	1,01910556	1,34569343	0,50268705	0,86761359
	0,00643001	0	0,80199531	1,46612068	0,50648498	0,99372058
	0,00319875	0,11465405	0,42220024	1,01507287	0,36765822	0,94806124
**DNMT3AB**	0,02361568	0,09444508	0,99199152	2,41709573	0,52523414	0,48349663
	0,01676957	0,26024275	0,65206766	2,52735837	0,62467015	0,63942591
	0,029517	0,09932451	0,33337285	1,84613481	0,58145328	0,56970362
**DNMT3BA**	0,00043153	1,16478793	4,93240973	1,58821936	0,14085246	0,28159727
	0,00047607	1,51471378	3,62933017	1,60154142	0,16455945	0,40538305
	0,00065101	1,41263908	3,35708432	1,47702294	0,18040207	0,32798759
**UHRF1**	1,21534217	1,43458301	2,1278371	1,01578924	0,0348178	0,5711331
	0,67480414	1,80659319	1,38623649	1,28233695	0,04656991	0,79579227
	0,96764529	1,22520454	1,23997943	0,92224983	0,04411081	0,63343631
**TET1**	0,0032537	0,20416573	1,33967772	3,36694754	1,72926172	0,88039209
	0,00232247	0,08267852	1,21431612	3,21493579	1,67047063	0,78726908
	0,00291597	0,09270208	0,7561546	3,17679948	1,54755775	0,72267375
**TET2**	0,00352881	0,16877158	1,25242432	3,27761328	1,41277071	0,86684258
	0,00107743	0,0737401	0,95529056	3,88527166	1,30976213	0,841509
	0,00188262	0,10449	0,44122864	3,19743093	1,14159519	1,00074444
**TET3**	0,00494553	0,02433131	1,38404544	3,58260578	0,96865103	0,6552052
	0,00446334	0,02320943	0,96081971	4,83352549	0,91726385	0,61833736
	0,00493571	0,01790248	0,27289956	4,29576217	0,95189842	0,73766066

4B. qRT-PCR analysis (aging tissue) – TBP normalized mRNA expression													
		**Week 5**			**Week 11**			**Week 15**			**Week 19**		
**DNMT1**	Liver	0,584936191	0,565055343	0,750097245	1,58606842	1,52467925	1,65496758	1,43234719	1,10149652	1,31961555	1,186292653	1,050597219	1,633389592
	Muscle	1,373923916	1,481400842	0,950530321	1,06687124	0,64311514	0,89236842	0,93975677	0,82481124	0,57502261	0,699631699	0,905055371	0,847532562
	Brain	1,199612303	0,991988457	1,147973101	1,18465924	0,8593074	0,86626498	0,7393532	0,76048691	0,85546417	0,578767781	0,852439222	0,9608171
**DNMT3AA**	Liver	4,586140536	3,536208829	7,027029508	5,57370712	6,13234973	6,20678698	2,06008891	3,02326642	3,02840433	2,229551265	1,926990852	2,249759339
	Muscle	2,021198487	1,255058263	1,076441491	1,02700512	1,03675565	1,09159911	1,38191131	0,85293417	0,79309722	0,485950312	0,756958304	0,729127248
	Brain	0,74239765	1,660781146	1,00704056	0,82879771	0,89272036	1,11406475	1,17444057	0,87365995	1,19217127	0,395778515	0,854730728	1,03278578
**DNMT3AB**	Liver	0,484737559	0,660539919	0,894330906	0,61314187	0,91129275	0,91544585	0,62345977	0,51809114	0,60566354	0,512176305	0,864320206	0,57485525
	Muscle	1,319781385	0,977068257	0,704190154	0,70526312	0,53098214	0,64427489	0,46358332	0,43703503	0,30384394	0,498890126	0,618942144	0,434009275
	Brain	1,752003748	1,013313918	1,22870621	1,04419386	0,9912002	0,76223159	0,75225875	0,65728278	0,71378047	0,571005287	0,655428821	0,662882177
**DNMT3BA**	Liver	1,373561219	1,187975554	1,536853599	0,93906744	1,65895489	1,65156836	1,29902222	0,47057645	0,62397753	0,556252436	0,885387654	2,212355972

	Muscle	2,116860006	1,647612918	1,584369955	0,70621093	0,56273161	0,88303757	0,77421888	0,8296364	0,50349303	0,44034334	0,882411775	0,85439592
	Brain	1,754737089	1,100851889	1,129118577	0,88351162	0,78733685	0,72585052	0,60578567	0,43468058	0,58681209	0,693074612	0,777697604	1,332650606
**UHRF1**	Liver	0,363766866	0,63995407	1,08270218	7,47827035	2,46754925	3,71412863	1,63507158	1,08293964	2,1372921	1,057585394	1,149703581	5,274500507
	Muscle	1,389050186	3,210509353	1,419894739	1,8113843	0,66448579	1,23418332	0,93835571	0,29999235	0,74864043	0,959556669	0,498326562	0,836139124
	Brain	1,556016856	0,95101819	1,035623574	0,79199243	0,64282185	0,62021703	0,50058091	0,42175576	0,48104107	0,483099475	0,824156108	1,196079614
**TET1**	Liver	0,715128129	1,412021263	0,906457737	0,72520631	1,07112284	1,2668235	0,71435351	0,80650658	0,60238024	0,851630296	0,752378679	0,911131657
	Muscle	0,943440836	0,787183735	0,776092431	0,880042	0,8701596	0,76859393	0,95927296	0,61865053	0,50410806	0,606152185	0,746775667	0,596637174
	Brain	1,284433427	1,319512534	1,109831144	1,28332656	1,13462312	1,00844694	1,61977643	1,09234556	1,24574208	0,775088007	1,168954865	1,168745117
**TET2**	Liver	0,756012933	1,581478292	0,896850809	1,20056223	0,55444828	1,24917328	0,9014198	1,12408984	0,82219069	0,828893147	0,737365196	0,894311283
	Muscle	0,841696038	0,493091718	0,99889653	0,93742536	0,85422941	0,77574458	0,92858523	0,68241495	0,60751376	0,46601606	0,482582299	0,726131724
	Brain	1,277787889	1,167117272	1,122109418	1,21362163	0,96256496	0,91745688	1,21066368	0,88597756	1,01184478	0,883019362	1,039554954	0,834936477
**TET3**	Liver	1,860457053	2,640697923	1,071885162	1,65932351	1,99685975	2,17600283	1,18182232	1,14818896	0,8642817	1,000824689	0,75396703	0,678787376
	Muscle	1,293873144	1,194896283	0,895326846	1,17817944	0,85061587	0,94263973	0,91713217	0,80189765	0,75513623	0,888791587	1,054938676	0,768436198
	Brain	1,707640376	1,391857532	1,345999238	1,2749017	1,29691031	1,15144128	1,20729897	0,91335097	0,99755901	1,079072884	1,202042392	0,938669151

4C. dot blot analysis (aging tissue) – blot intensity normalized to week 5, replicate 1

		Week 5			Week 11			Week 15			Week 19		
**5mC**	Liver	1	1,029740846	1,10650081	0,798727466	0,735887538	0,981578357	0,647527997	0,660772358	0,836382791	0,691796599	0,498679188	0,662485439
	Muscle	1	0,682255507	0,942118352	0,875793053	0,49066516	0,72376465	0,668465085	0,482465519	0,597516004	0,555815534	0,387698454	0,378677331
	Brain	1	1,134050215	1,192804985	0,901950476	1,023839854	1,24901181	0,97272775	0,973140157	1,312198023	0,75956942	0,810793566	1,049667499
**5hmC**	Liver	1	0,852479184	0,996387523	0,770128953	0,660583309	0,812529954	0,844484724	1,17312832	0,982602018	1,068767417	0,839373345	0,791098693
	Muscle	1	0,722686418	0,861599877	1,124770263	0,893018083	1,05481406	1,091927511	0,877585609	0,853622884	0,810971304	0,72881806	0,642463892
	Brain	1	0,992383596	0,863496325	0,937678078	0,968281717	0,928299762	0,855516837	0,92866189	0,729018319	0,740380614	0,795114159	0,73184653
